# The Responsibility of the Dentist and Physician in Regard to Mouth Infections and Their Relation to Constitutional Effects*This interesting paper was read before the section on Stomatology at the recent meeting of the American Medical Association and is reprinted from the Journal of the A. M. A., of October 4th, 1913. The Author very wisely intends to present to the Medical Profession some evidences of systemic infection from oral diseases, and he makes a good statement of his case, which it will be well for all dentists to take into account in their diagnosis and treatment of these oral diseases.—*Editor Register*.

**Published:** 1913-11-15

**Authors:** Thomas B. Hartzell

**Affiliations:** Minneapolis, Minn.


### THE RESPONSIBILITY OF THE DENTIST AND PHYSICIAN IN REGARD TO MOUTH INFECTIONS AND THEIR RELATION TO CONSTITUTIONAL EFFECTS *

THOMAS B. HARTZELL, D.M.D., M.D., MINNEAPOLIS, MINN.

The human mouth is the largest portal through which infectious micro-organisms may enter the body. Almost all varieties of pathogenic bacteria have been found in the human mouth. The gastric juices afford little protection against them. Charles Mayo[1] says:

---

* This interesting paper was read before the section on Stomatology at the recent meeting of the American Medical Association and is reprinted from the Journal of the A. M. A., of October 4th, 1913. The Author very wisely intends to present to the Medical Profession some evidences of systemic infection from oral diseases, and he makes a good statement of his case, which it will be well for all dentists to take into account in their diagnosis and treatment of these oral diseases.—*Editor Register.*

1. Mayo, Charles H.: Constitutional Disease Secondary to Local Infections, Dental Review, April, 1913, p. 281.

Microscopic examination of gastric extracts made by Smithies from 2,406 different individuals with "stomach complaint" (dyspepsia, indigestion and the like) showed that irrespective of the degree of acidity of such gastric extracts, bacteria were present in 87 per cent. Morphologcally cocci and diplococci were present in 83 per cent.; short and long rods (often of the colon group) in 58 per cent.; typical streptococci and staphylococci in 17 per cent.; and *Leptothrix buccalis* in 24 per cent. In fifty-four cultural studies of saliva from "dyspeptic" patients, streptococci and staphylococci were demonstrated in over 80 per cent., bacilli in 66 per cent., and *Leptothrix buccalis* in more than 14 per cent. Comparing these figures it would appear that the common forms of pus-producing organisms (streptococci and staphylococci) have their proliferation retarded in gastric juice, but that bacilli (often of the colon group) as well as *Leptothrix buccalis* thrive in the stomach.

There is no protection to the organism where an actual lesion exists except that afforded by the leukocytes. The leukocytes in time are overcome in their effort to oppose the destructive effects of micro-organisms. The almost universal presence of small lesions contiguous to the teeth which afford foot-hold for pathogenic bacteria which by any chance gain entrance to the mouth, makes it doubly worth while to study the paths which the bacteria must travel to gain entrance into the circulation. A brief glance at the tissues in question will serve to accentuate the importance of a closer study of mouth infections.

ANATOMIC AND PHYSIOLOGIC CONSIDERATION

The teeth occupy sockets in the alveolar bone, which is built up around the teeth as they erupt. This bone is of a loose, porous character and has very thin edges; on the whole, an insecure frame-work to support the great stress of mastication, amounting to hundreds of thousands of pounds in the course of a year, which the teeth must bear. The teeth are suspended in their sockets by Sharpey's fibers, yellow elastic tissue, which is a ligament of suspension, plentifully supplied with blood through the alveolar process and also through the mucous membrane, the vessels dipping down into the ligament of suspension over the edge of the socket. In this membrane are resident certain glandular structures first described by Black. These glandular tissues are in direct continuity with vascular channels leading out into the mucous membrane and alveolar process. Therefore, any infectious material on the teeth themselves or accumulated between the teeth, at the free margin of the gum, has an uninterrupted avenue of entrance into the deeper structure of the jaw. The continuity of the vascular channels leading from the periodontal membrane into the deeper structures is readily demonstrable by the injection of pure oxygen gas under the gum margin by means of a blunt-nosed needle. In this way one can trace these vascular channels through the alveolar process for considerable distances. Therefore, the pyorrhea pocket, which is an ulcerating infected surface full of micro-organisms, or the alveolar abscess, which usually occurs in the apex of the socket around the root-end, are both capable of introducing into the circulation the severest types of infection. If we bear in mind the conical nature of the tooth-sockets and the fact that the tapering roots filling these conical sockets support structures which in every twenty-four hours of the ordinary individual's life may bear a stress of from five to ten thousand pounds, one readily comprehends how infections in the periodontal membrane or about the apices of the tooth's root are forced into the general circulation and lymphatic channels, leading from the teeth in their sockets. The direct lymphatic drainage of the tissues contiguous to the teeth is the most perfect in the whole body, affording direct access to the deeper structures of the neck, maxmilla and mediastinum, thereby making it possible for micro-organisms, introduced into the tissues through a pyorrhea pocket or apical abscess; to travel uninterruptedly into the deep structures of the body. Not only do the lymphatics afford direct access for micro-organisms to the circulation, but the rich plexus of blood-vessels supplying the periodontal membrane and alveolar process invariably suffer from the destructive activity of pus-forming bacteria, opening the blood-vessels themselves to the entrance of germs which are injected into the broken ends of these vessels by the powerful stress of occlusion during the act of mastication, thus making the spread of bacteria in the circulation a certainty. The elastic nature of the periodontal membrane makes it possible for the teeth to bear the shock of mastication for many years longer than they possibly could were they ankylosed into the bone. But this same elastic quality in the periodontal membrane favors the introduction of infection. For, if there is infectious material around a root end, or about the side of a root in a conical socket, and a weight of from 10 to 250

pounds is applied to the occlusial end of such a tooth, the elastic quality of the membrane permits the tooth to sink down in its socket so that the root acts as a plunger, injecting the infectious material into the surrounding tissues.

The teeth themselves afford many square inches of surface for bacterial protection and growth, as they measure in a full denture about 20 linear inches in circumference, and, where destruction of the bony socket attains a depth of an eighth of an inch, we have the equivalent of $2\frac{1}{2}$ square inches of cultural space beneath the gum margin. The necks of the teeth above the gum margin, protected from attrition by the broad bell-shaped occlusal portions of the teeth, afford easily from $2\frac{1}{2}$ to 3 square inches more space on which bacteria may accumulate. This bacterial accumulation on the necks of the teeth is rarely noticed or appreciated, unless some special method of staining is adopted to reveal the presence of micro-organisms. If one desires to appreciate the enormous possibilities for bacterial growth on the necks of the teeth, one should apply a stain which will at once bring into view the bacterial coat always present on these surfaces.

### STAINING METHOD.

The best disclosing stain for revealing bacterial presence on the tooth's surface is iodin, 45 grains, zinc iodid and potassium iodid, of each 15 grains, water and glycerin, of each 4 ounces.

A cotton sponge dipped into the disclosing stain and applied to the necks of the teeth instantly brings into view a heavy brown coat on the teeth, which, before the application of the stain is made, may seem white and clean.

I advise physicians and dentists who are in doubt as to the cause of the marginal inflammations of the gum and underlying bone to apply always the above disclosing stain, after which the mouth should be rinsed with water, which will leave in clear relief whatever bacterial coat the teeth may at that time bear. If this practice is followed, the observer will be impressed with the constant presence of areas of inflammation and deep pockets of destruction in the alveolar process and bone contiguous to the necks of those teeth which bear the heaviest bacterial coat.

### PATHOLOGY OF MOUTH INFECTION.

The enormous surface for bacterial growth on the teeth and the protected areas between the teeth and the deep pockets which form in the presence of the heavy bacterial coat which these protected surfacs usually bear, give rise to a multitude of organisms, which of necessity pass into the stomach and bowels, and frequently alter, by their products and presence, the whole chemistry of digestion, so that an infected mouth may produce four distinct pathologic effects as follows:

1. That produced by the dissemination of bacteria through the medium of lymphatic drainage.
2. That produced by bacteria through the open blood-vessel.
3. That damage sustained by the individual through the change in the chemistry of digestion caused by bacterial poisons.
4. That produced by a general bacteriemia which not infrequently is a direct result of the dissemination of bacteria in the blood-stream.

I would not contend for a moment that all the ills to which human flesh is heir may be traced to mouth infections, but I have observed many cases of general infection of different characters which have been traced directly to mouth infections. The ordinary mouth infection may result in the loss of the teeth only, and the general harm resulting from such infection may be only slight. Statistics gathered by me show that there is about one case in every ten in which severe constitutional lesions occur, lesions which are usually overlooked both by physician and dentist, traceable to the mouth. I present herewith the analysis of one thousand and twenty cases of mouth infections:

SUMMARY OF FINDINGS IN 1,020 CASES OF MOUTH INFECTION

It is to be remembered that in the locality in which this experience has been gathered, the population is a young and vigorous one, as compared to the population of older countries, and the death-rate is lower per thousand on account of this fact. Here, the proportion of aged people to people under 40 is much less than that of older localities. Of the one thousand patients observed, few were bed-ridden. In the following deductions, these facts were kept in view. Over half, or, to be accurate, 600 seemed to be absolutely healthy and normal in every way except in the matter of teeth and gums. Caries was more or less prominent in the whole number, and marginal infections, with a greater or less destruction of alveolar process, were common to all of them. There were no marked physical symptoms of ill health, aside from indigestion or mild forms of gastric irritation. As a rule, on correction of the mouth infection, all these symptoms disappeared. Of the remaining four hundred, 300 gave evidence of more severe physical disturbances, though not severe enough to warrant sending them to a physician, or a more careful scrutiny of their habits of life than treatment of the mouth lesions and the proper rebuilding of the masticating mechanism. In the remaining 120 cases a great variety of physical ailments was observed, and physical examinations were made. The following lines of inquiry were noted and any facts which such inquiry elicited were tabulated: occupation, habits, general systemic condition, temperature, urinalysis, oral history, condition of the nose and throat, quality of the saliva, occlusion, the presence or absence of deposits on the teeth, and the character of such deposits, if present. Note was also made of undue stress, loss of function, prosthesis existing, and the prosthesis needed. The urinalysis involved a consideration of the total bulk of urine in twenty-four hours, its specific gravity, and examination for albumin, sugar, and a search for casts. The physical examination further sought to reveal or explain the systemic conditions which were found to exist in each individual case.

In eighty of these cases, the disturbance ranged over the entire alimentary tract, some patients being chronic sufferers from constipation, others from the opposite condition, namely, looseness of the bowels and diarrhea, all evidencing more or less malnutrition. Fifty had periodic and severe migraine, and the whole group suffered from more or less nervous irritation. Forty were of the extremely neurasthenic type. A considerable number of this group of 120 were also sufferers from chronic abscess. To be accurate, thirty-five had chronic apical abscess, and twenty had severe pericemental abscess as well. Sixty of them gave a history of rheumatoid pains, called by some of them neuralgia, by others rheumatism. In eighty cases, no kidney involvement or abnormal change in the urine was observed. Without exception all were benefited by treatment, showing a marked recession of constitutional symptoms on removal of abscessed and hopelessly loose teeth, and on surgical treatment of those teeth remaining which were worth treatment, followed by careful postoperative care of the mouth. Of the remaining forty cases, all showed greater or less kidney involvement, ranging from a trace of albumin to a considerable amount, with casts. Ten showed the presence of sugar. Twenty showed specific gravity of urine ranging from 1.020 to 1.035. Twenty revealed decreased specific gravity, with a corresponding increase of bulk in the twenty-four hours. Three were suffering from septicemia, and died of septic endocarditis. One died of septicemia without heart involvement. This was a recent case, in which bacteria were found in the blood-stream on culture, which bacteria were identical with those found in the pockets about the teeth, and also in an area of necrosis located about the two upper central incisors. Of this group of forty, twenty patients had pronounced joint involvements, ten of which were distinctly improved by treatment; one might, in fact say, cured. Five were improved and five not benefited. Of these forty cases, nineteen patients showed albumin in the urine before the mouth infection was attacked, which cleared up after the stamping out of the mouth infection. One of these patients had been suffering from ulcer of the stomach, the diagnosis of which was made by an exceedingly competent medical adviser, and was of two years' standing, though believed to be much longer. In this particular case, albumin was present in the urine. This patient suffered an intractable constipation, accompanied by a marked failure of stomach digestion. He yielded to treatment readily and after a period of two months gained in weight over 20 pounds, and lost all evidence of tenderness in the abdomen over the area where stomach ulcer was believed to exist.

A recent case is of great interest. The patient, a banker, suffered severe pain in two of the lumbar vertebrae, accompanied by pain and soreness in the wrist, elbow and finger joints. He made no material improvement until several abscesses, teeth, and pus infections in pyorrhea pockets were eliminated, after which he made a fairly rapid and satisfactory recovery. This patient was a man 58 years of age, and the total period of his incapacity from business had been eight months. For four months of this time his suffering was exceedingly acute, being bed-ridden. On account of the great tenderness in the vertebra, he was allowed to turn over in bed only once in twenty-four hours. Tuberculosis was not present. None of the symptoms bore out such a diagnosis.

In another case, the patient, a man of 62 years, suffered from a persistent and destructive inflammation of his left eye, having continuous pain for months, gradually losing the sight of the eye, and suffering intense pain radiating from the eye to the occipital region and down into the neck and shoulder. An abscess was finally located in the lower mandible near the mental foramen, involving the inferior dental canal. The opening of this abscess resulted in complete freedom from pain and a fairly rapid recovery after an illness of five months. The patient regained his bodily strength and vigor, but never regained the sight in his left eye.

There really seems to be no function or tissue of the body which may not be reached by infections occurring in or originating through lesions in the oral tissues. It seems eminently proper, therefore, that physicians should scrutinize more closely the mouths of their patients in all their routine examinations; insist on radiographic examination to discover impacted and diseased teeth; and insist on the extraction of diseased teeth, unless the patient is in the hands of a competent dentist who comprehends the vital relation which diseased and impacted teeth bear to the general health of the individual.

